# Unequal allocation between male versus female reproduction cannot explain extreme vegetative dimorphism in *Aulax* species (Cape Proteaceae)

**DOI:** 10.1038/s41598-022-05558-4

**Published:** 2022-01-26

**Authors:** Jeremy J. Midgley, Michael D. Cramer

**Affiliations:** Department of Biological Sciences, University of Cape Town, P. Bag Rondebosch, Cape Town, 7701 South Africa

**Keywords:** Ecology, Evolution, Plant sciences

## Abstract

Female plants not only flower but also produce resource-rich seeds, fruits, and cones. Thus, it is generally considered that female plants allocate more resources to sexual reproduction than male plants and that this allocation difference can explain vegetative dimorphism, such as greater leaf size in females. We found significant sexual vegetative differences in the dioecious and serotinous species*, Aulax umbellata* and *A. cancellata*. Plant height, annual branch length and canopy spread were greater in males whereas leaf size, branch thickness and branch number were greater in females. Sex ratios and basal stem area were, however, equal in the sexes. Equal sex ratios imply equal allocation to sexual reproduction and equal stem areas imply equal resource use and biomass, and thus allocation to vegetative growth. Given equal allocation to reproduction and resource use, we suggest that the vegetative dimorphism is driven by intra-male-competition to be more visually conspicuous to pollinators. This implies that plant architecture is both a vegetative and a reproductive trait.

## Introduction

Female plants must not only allocate resources to flowering but also to producing seeds as well as fruits and/or cones. This suggests that the costs of reproduction are higher for female than male plants, or for female function of hermaphrodite plants. Some dioecious plants (i.e. separate male and female plants) are vegetatively very different (i.e. dimorphic) between the sexes, such as females having larger branch and leaf sizes. Differences between male and female resource allocation to reproduction and the possible consequences of this for vegetative dimorphism in dioecious plants, is a central issue in plant evolution but it is a controversial and difficult topic^1,2^. In the most highly cited paper on this topic, Obeso^1^ notes it is practically impossible to measure the direct costs of male and female allocation to sexual reproduction. For example, most vascular plant species (about 95%) are hermaphrodites which makes measuring direct allocation by the two sexes, difficult. Thus Paterno et al.^3^ used an indirect allometric method to measure sexual allocation in hermaphroditic inflorescences and concluded that larger flowers represent greater relative allocation to male function.

The problem of shared sexual allocation to inflorescences is avoided in dioecious plants making them important tests for ideas of sexual allocation in plants. However, they are both rare as species and as individuals and are typically large, forest trees. For example, there are relatively few dioecious individual trees in the very large Barro Colorado forest tree data set^4^. Again, this large size makes direct measurement, such as of allocation to reproductive structures, difficult. The Cape Floral Region is a useful place to investigate sexual allocation in plants and its consequences, because dioecy is relatively common and vegetative dimorphism between the sexes can be extreme. Also, Cape plants are amenable to research being short (about 2–5 m), rapidly mature and short-lived (about 5–20 years). Thus, the large (about 85 spp.) Cape genus *Leucadendron* (Proteaceae) is probably the most researched genus globally for male and female differences^5–14^.

Even in these dioecious plants it is difficult to directly measure allocation to male and female function because of the difficulty of finding a common currency to compare allocation. For example, comparing allocation differences in attractiveness, nectar, seeds, pollen, cones and fruits and differences in the timing of producing these structures^1^. In *Leucadendron* males are generally more visually attractive than females. This is achieved by the loss of photosynthetic capacity in floral leaves and bracts^12,15^. It would be difficult to directly compare this photosynthetic loss in males, with for instance, female allocation to cones and seeds. Despite the difficulties in directly measuring and comparing allocation to reproduction, the consensus is that female allocation to sexual reproduction typically exceeds male allocation^1^, including in *Leucadendron*^2,9^.

Greater female allocation to reproduction is one of the suggested reasons for vegetative dimorphism between the sexes^2^. The three main hypotheses for sexual vegetative dimorphism are (i) greater female sexual resource allocation requires this to be balanced by having a more efficient physiology (resource use efficiency hypothesis), or (ii) greater female allocation requires females to be in the more optimum habitats (the sexual site dimorphism hypothesis) and this facilitates vegetative differences, such as larger female leaves in the more mesic habitats. Finally, (iii) vegetative dimorphism may be a consequence of selection on reproductive traits (reproductive traits hypothesis). In support of the resource use efficiency hypothesis in *Leucadendron*, Harris and Pannell^9^ argue that supplying water to live, closed cones in the canopy of serotinous *Leucadendron* females is a form of maternal care that non-serotinous species and males do not incur. To keep these cones from opening they need always to be hydrated and therefore serotinous females need to be more efficient in their water use than their males. They argued that fewer and thicker branches in females provides a hydraulic advantage. However, the data in Midgley^8^ and Roddy et al.^14^ showed no support for sexual differences in water use efficiency. Clearly, there are opposing views as to whether females allocate more to reproduction than males and whether females are eco-physiologically more efficient than males.

The sexual site dimorphism hypothesis has not been tested for *Leucadendron* presumably because males and females co-occur on a small spatial scale^16^ but is tested in the present analysis of *Aulax umbellata* and *A. cancellata*. In support of the reproductive trait’s hypothesis, it was argued^5^ that in *Leucadendron*, vegetative dimorphism is an allometric consequence of selection for smaller male inflorescences. Smaller inflorescences are then associated with more, but narrower, stems and thus smaller leaves via Corners Rules^5^. Besides the evolutionary relevance for understanding sexual differences in allocation, it may also have conservation implications. For example, Hultine et al.^17^ argued that dioecious plants are under more threat than hermaphrodites because dioecious females are presumed to allocate more resources to reproduction than males. As global change progresses, females may suffer greater mortality and thus dioecious populations may have lower reproductive potential if they become more male biased.

One way around the measurement problem of determining direct allocation to sexual reproduction is to use indirect methods based on trade-offs^1^ such as the influence of allocation to sexual reproduction, on sex ratios and sizes of co-occurring male and female plants. If for example, males allocated less to reproduction than co-occurring females, they should be relatively larger or live longer and this would impact size and sex ratios, especially as plants age and competition intensifies.

The Cape is uniquely suitable to consider allocation differences between the sexes because populations of dioecious Cape species are often large (> 1000’s of plants ha^−1^) and with males and females co-existing at a fine spatial scale. The Cape Proteaceae grow in a stressful summer dry Mediterranean climate with nutrient-poor soils^18^. This provides strong selection on reproductive allocation to seeds (such as large size and high nutrient concentrations) to produce seedlings large enough to survive their first summer. The Cape Proteaceae are strongly fire-adapted. For example, many species are serotinous (canopy storage of seeds in live, closed cones which mainly open after fire)^19^. This too requires high female sex allocation to maintaining cones in the canopy. Most Cape Proteaceae species are post-fire re-seeders^19^ in that all plants die in fire. This results in single-aged populations of single-stemmed non-clonal individuals; adults die in fires and dense patches of seedlings establish in the first winter after the fire and die in the next fire. Co-occurring males and females have the same age and thus differences in size or sex ratios will mostly reflect allocation differences and competition rather than age or habitat. Also, because seedlings in the Cape grow up in an open post-fire environment, woody plants do not need to allocate specifically to height growth, to achieve full light. They are in full light their whole lives and therefore any sexual architectural differences do not reflect differences in habitat shadiness. Here we focused on *Aulax umbellata*, but also present sex ratios and size metrics for the congeneric *A. cancellata*. These are two common, single-stemmed strongly serotinous Cape species in the Proteaceae which are highly vegetatively dimorphic. Although both *Leucadendron* and *Aulax* are dioecious, a rare trait in the family, this represents independent evolution as the two genera are not close phylogenetically^20^. We test the hypothesis that vegetative sexual dimorphism in *Aulax umbellata* and *Aulax cancellata* can be explained by differences in allocation to growth. We predicted that co-occurring males and females would occur in equal sex ratios and be equal in size due to equal growth, despite vegetative dimorphism.

## Results

### Size and sex ratios

**Figure 1:**
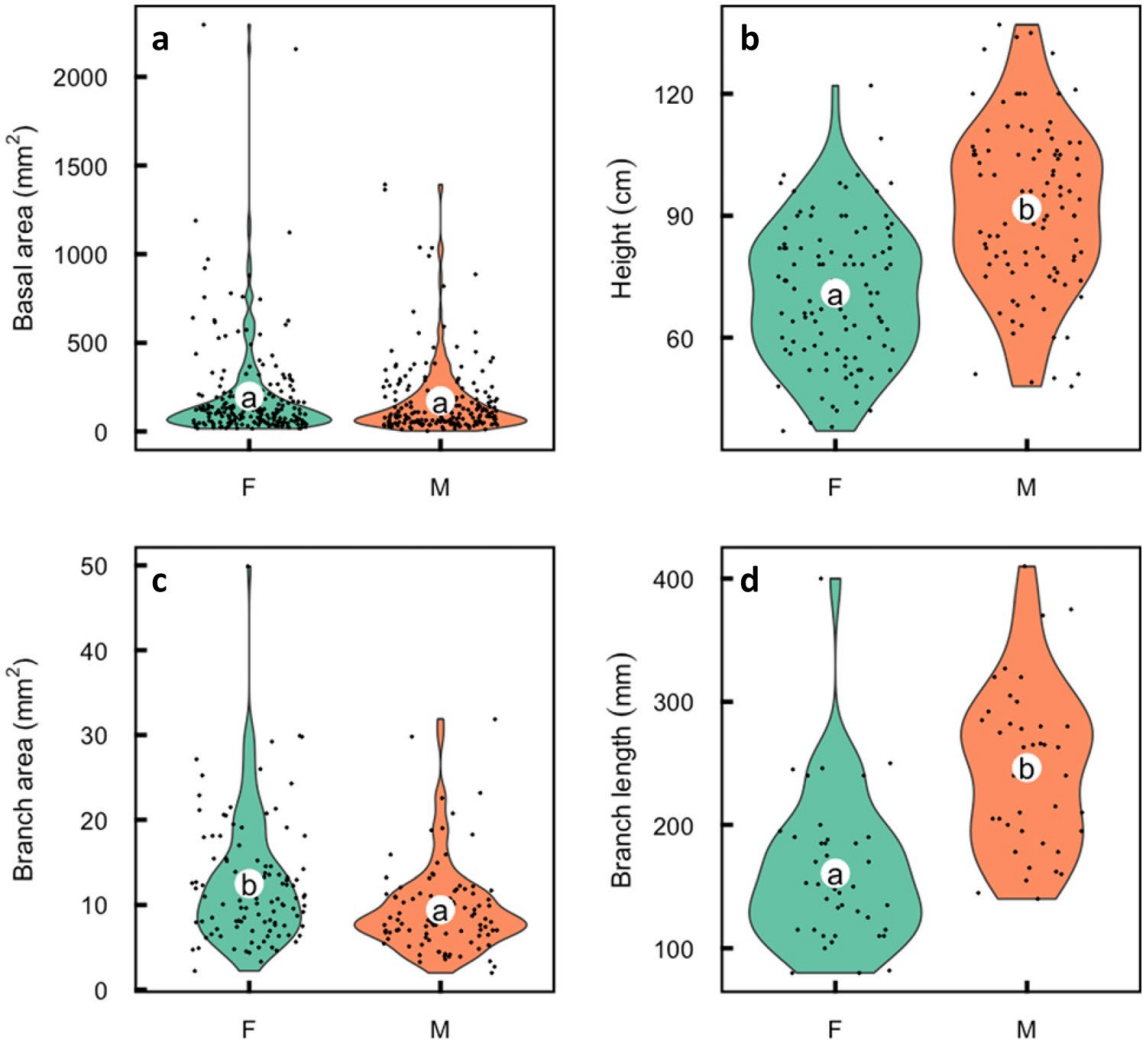
Violin plot comparisons of males and females of *Aulax umbellata*. White circles represent means, and with different letters indicating significant sexual differences (*p* < 0.05) in (**a**) stem basal area, (**b**) plant height, (**c**) branch stem area and in (**d**) branch length. Points represent the individual data points with jitter applied to the distribution along the x axis.

There is relatively high disparity in size (individual basal area) in all stands; Gini coefficients for *A. umbellata* at site 1–4 were 0.37, 0.45, 0.34, 0.52, respectively and that of *A. cancellata* was 0.30. This is evidence of competition producing few, large “winners” and many, smaller “losers” especially in the older (sites 2 and 4) post-fire stands. Despite this inequality there is no difference in mean basal area between the sexes (*p* < 0.203; Fig. 1a). Male *A. umbellata* are about 30% taller than females (*p* < 0.001, Fig. 1b). Mean female height at site 1 and site 3 are 65.3 cm and 76.1 cm, whereas male heights are 86.9 cm and 96.7 cm respectively and thus males are taller for a given basal diameter.

Sex ratios are equal with *A. umbellata* male:female 47/58 (site 1), 49/53 (site 2), 48/57 (site 3) and 51/64 (site 4; binomial test n.s.) and 163/162 (n.s.) for *A. cancellata*. Sex ratios did not change with an increase in size inequality. Males and females were well mixed especially in the older stands (runs tests; site 1 *p* = 0.24, site 2 *p* = 0.84, site 3 *p* = 0.24, site 4 *p* = 0.85).

### Canopy differences

**Figure 2:**
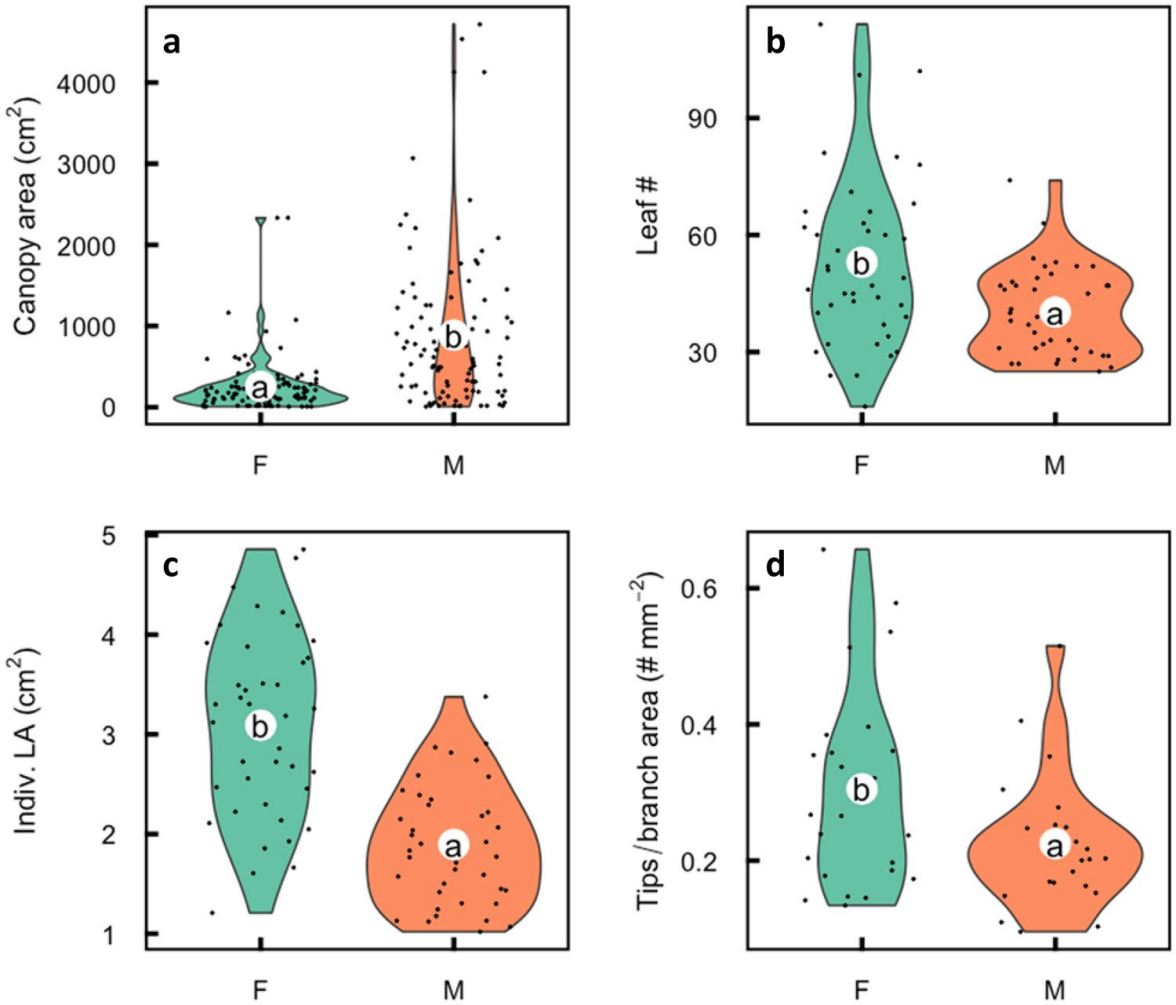
Violin plot comparisons of males and females of *Aulax umbellata*. White circles represent means, and with different letters indicating significant differences (*p* < 0.05) in (**a**) canopy area, (**b**) individual leaf area, (**c**) leaf number per branch and (**d**) branch tips per unit branch diameter, resulting from post-hoc Tukey tests. Points represent the individual data points with jitter applied to the distribution along the x axis.

Males achieve canopy differences by having significantly thinner (*p* < 0.001; Fig. 1c) but longer, annual branches (*p* = 0.001; Fig. 1d) compared to the females. Male canopy spread is about 3 times that of females (*p* < 0.001; Fig. 2a). Mean female canopy spread at sites 1 and 3 is 48.3 and 49.6 cm^2^, whereas males were 135.7 cm^2^ and 139.6 cm^2^ respectively. Males had significantly smaller leaves than females (1.9 vs. 3.1 cm^2^; *p* < 0.001; Fig. 2b), fewer leaves per branch (Fig. 2c) but leaf SLA did not vary between the sexes (4.7 vs. 4.8 m^2^ kg^−1^; *p* = 0.50). Male branches were longer for a given diameter than were female branches. Sampled female branches had mean stem area of 68.7 mm^2^, length of 45.4 cm and 19.6 branch tips, whereas males were 54.1 mm^2^ in stem area, 66.4 cm in length and had 11.9 branch tips. In a linear model with branch length, branch stem area and sex to predict the number of branch tips, branch length and branch stem area were highly correlated and thus only branch area was retained in the final model. The final model with branch area and sex had an adjusted R^2^ of 0.28 and *p* < 0.001, with sex as a significant predictor (*p* = 0.004; Fig. 2). Seed set was above 85% (mean floret number of 11.9. and mean seed number 10.3). In *A. cancellata,* female leaves were longer than males (mean 8.1 cm vs. 5.5 cm; *p* < 0.01), mean male branch length was longer than of females (10.1 cm vs. 5.9 cm; *p* < 0.01), but basal area (female 47.9 mm^2^, male 44.2 mm^2^, *p* = 0.26) did not differ significantly.

## Discussion

One to one sex ratios in mature plants, such as we have found, indicates situations where maternal plants can achieve equal fitness through either their male or female off-spring (Fisher’s principle^21^). Equal mean fitness of males and females implies equal costs of reproduction. This is because the genetic benefits of sexual reproduction are equal (roughly 50:50 contribution to off-spring genome) between the sexes and therefore the costs must be equal.

Given the sexes of both species have the same mean basal area, they likely use a similar amount of soil resources and have same biomass. Given the above argument for equal allocation to sexual reproduction, equal resource use indicates equal allocation to vegetative growth. Previously, it was argued^13^ that in the Cape because co-occurring males and females are the same post-fire age, there is no advantage for either sex to allocate differently to reproduction to grow larger or live longer. This is because typically the next fire will simultaneously terminate the lives of both males and females. There is thus no future opportunity to benefit from delayed allocation to reproduction in favour of vegetative growth. There is no reason for, nor evidence of, unequal allocation to vegetative growth or reproduction. Based on nearly identical SLA, the leaves of the sexes are physiologically similar. The resource use hypothesis can thus be rejected as a cause for vegetative dimorphism, as can the sexual site dimorphism hypothesis because males and females co-occur. Males achieve greater height by having fewer, longer, and thinner branches, each supporting fewer, smaller leaves. This vegetative dimorphism is curious as there is no physiological benefit in males being taller, such as access to greater light, because plants grow in full light their whole lives and have sparse canopies.

Despite the bright yellow colour of male inflorescences, only a limited insect fauna visits *Aulax* inflorescences^22^. We suggest that male-male competition for pollinators has led to selection for greater attractiveness and conspicuousness (height, and canopy size) amongst males. Females produce little reward to insect pollinators, having neither nectar^22^, nor pollen. Nevertheless, seed (fruit) set is extremely high in the females with > 85% of female florets setting seeds and 57% of these seeds are viable. Seed set in *Leucadendron* is similarly high and both are much higher than in hermaphrodite Cape Proteaceae^12^. In *A. umbellata* low female conspicuousness, attractiveness, and fewer rewards are nevertheless sufficient for high seed set; implying many visits by a pollen carrying visitor per inflorescence. In accordance with Batemans Principle and the sexual selection and dimorphism it predicts^23^, male plants are more conspicuous and attractive than females to attract as many pollinators as possible. Greater plant height and canopy spread of males is thus also an allocation to reproduction. This implies that in these species it is not possible to determine the extent to which the sexes allocate separately to vegetative growth or reproduction, as they are not separate allocations.

**Figure 3:**
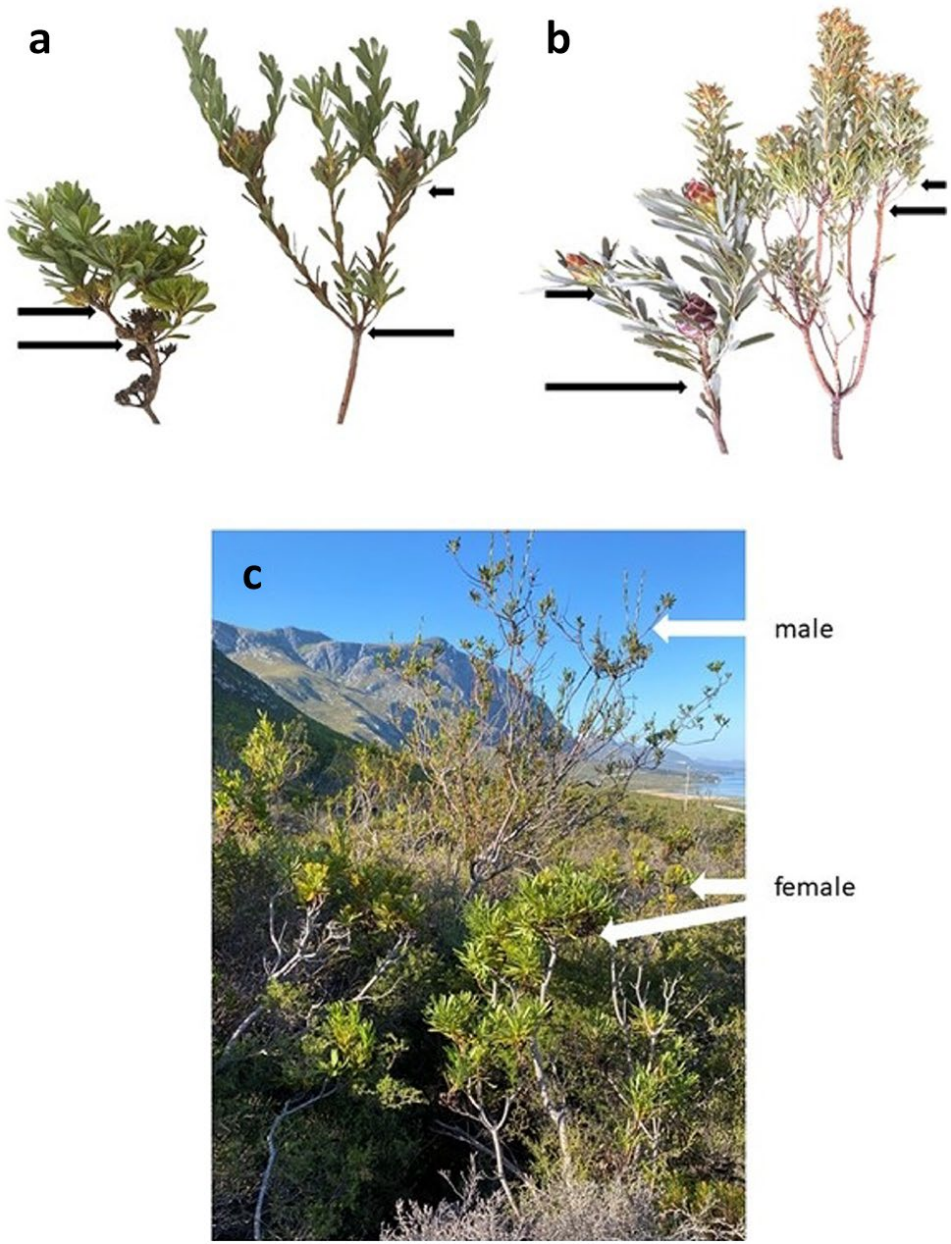
Male (right) and female (on the left, with cones) branches of similar thickness, (**a**) *Aulax umbellata* and (**b**) *Leucadendron rubrum*. Note male annual branches (between the arrows) are relatively longer, thinner, and fewer compared to females in *A. umbellata*, whereas male branches are shorter, thinner and more numerous in *L. rubrum*. The photograph of *A. umbellata* male and female plants near site 1 of the same age but vastly different size and architecture (**c**) showing short, more highly branched, and compact females in the foreground and taller spindly males in the background.

Harris and Pannell^9^ linked variation in canopy architecture to the requirements of females for resources to support the serotinous cones. They devised a ramification index based on the slope of the correlation between stem area against distance down the stem as a measure of branching. Their index is based on their stated assumption that branches vary only in number and thickness, not length. This assumption is violated in *A. umbellata* and *A. cancellata* as males have fewer, thinner but longer branches per unit length or stem area, than females. This means that males have lower ramification than females, which is the opposite of the case of *Leucadendron* and thus to their prediction that serotinous females should have relatively fewer branches than males. In *Leucadendron* the equal branch length assumption is also violated; males have shorter, thinner but more branches than females (Fig. 3). Also, Harris and Pannell^9^ did not define what a branching event is in terms of plant hydraulics. The relevance of this is that stem to leaf is a much more common hydraulic branching event than from stem to stem and stem to cone. In this respect, *A. umbellata*, females have more and larger leaves per branch than males (Fig. 2b,c). Also, the female cones in *A. umbellata* are each made up of about 10 shortened branches^22^, which we did not consider in our measurement of branch tips. Females in *A. umbellata* are thus much more highly branched (ramified) than males, in both stems and leaves, and therefore contradict the hypothesis of Harris and Pannell^9^. Furthermore, mature serotinous cones use little water in comparison to leaves or young cones^24^. Thus, lower ramification in females and associated physiological benefits do not explain vegetative dimorphism in serotinous *Aulax* species.

Finally, the interest of botanists with total male versus female differences in reproductive allocation is not shared by zoologists. For example, whether total allocation to sexual reproduction by males is different to females is not mentioned as a relevant topic in West^25^. This is because the typical 1:1 sex ratio in animals automatically implies equal lifetime allocation to reproduction by the sexes, whether there is maternal or paternal care or not. Sexual allocation in animals is not just allocation to the genitalia or off-spring, it includes all organs, as well as a host of sexually selected traits such as colour, ornaments, and behaviour. Also, allocation differences between the sexes in most animals would again be largely impossible to measure directly and to compare. Take elephants; the costs seem similarly as unequal between the sexes as in serotinous plants. The male merely contributes a haploid gamete (= pollen grain), whereas the female has pregnancy, lactation (= seed and cone formation) and several years of maternal care (= serotiny). The male however must produce and carry tusks, fight for access to females and many males may have zero fitness. These differences make allocation to sexual reproduction impossible to calculate directly in elephants, but 1:1 sex ratios would imply equal lifetime allocation to reproduction. Also, elephants have behavioural mechanisms to reduce conflict between males and females. Where male and female plants co-occur, they cannot avoid intersex competition, and this prevents one sex from being vegetatively superior to the other. For example, if the branch/leaf size of one sex was superior, then natural selection could not stop the other sex from converging on this size, unless it negatively affected that sex’s reproduction.

The modular nature of plants gives the impression that allocation to vegetative and reproductive growth are non-overlapping, but as we have argued for *A. umbellata* and *A. cancellata* the architecture is also an allocation to reproduction. Corner’s Rule (the correlation between leaf and inflorescence size with branch size and frequency) applies to many plant species^5,26,27^. It applies in *A. umbellata* and *A. cancellata* where smaller leaf size in males correlates with narrower stem size. Correlations between leaf, branch and inflorescence size and the resulting architecture, indicate that they are all simultaneously both a reproductive and a vegetative trait. We suggest that there is the expectation of equal allocation to reproduction by both sexes in dioecious and out-crossed hermaphrodite plant species, as it is expected in out-crossed bi-parental animals. Vegetative dimorphism in plants therefore in part results from sexual selection, as it does in animals. However, in plants the sexual selection is via the pollinators and thus pollinator behaviour will explain why some species are dimorphic and others not.

## Methods

### Study sites and species

*Aulax umbellata* and *A. cancellata* are two very common, non-threatened, easily identifiable (http://www.proteaatlas.org.za/feather.htm) members of the Cape Proteaceae. We thank the land managers for permission to sample and collect these two species. To test for sexual differences in size and frequency, we sampled all individuals of *Aulax umbellata* rooted within 1 m of either side of several 50 m transect through four populations. Cape Proteaceae plants produce one year of growth between branches, or inflorescences, or leaf scars and this is widely used to age plants or branches. We sampled *A. umbellata* in two areas about 25 kms apart; Hermanus (34 24 38.20 S; 19 18 18.80 E) and Kleinmond (34 19 49.78 S; 19 01 59.89 E) each with a relatively young post-fire stand (> 6 + years based on node counts, sites 1 and 3) and an older stand (> 12 + years based on node counts, sites 2 and 4). We focussed on *A. umbellata* but also sampled one stand of *A. cancellata* in Kogelberg Nature Reserve (site 5, 34 18 27.19 S; 19 00 00.68 E) in a single 30 m by 3 m plot to corroborate the *A. umbellata* results. Transects and plots were in homogenous habitats (flat, no obvious soil or moisture status changes).

### Size and sex ratios

To measure the size of individuals we used basal diameter because it directly correlates with the amount of water and nutrients moving up a stem^28^ as well as plant biomass^29^. Using digital callipers, we measured basal diameter of at least 100 individuals at all 4 sites. We were able to sex all individuals at these sites. At both younger sites (1 and 3) we additionally measured plant height, maximum canopy diameter and canopy diameter at right angles to this, as well as the length and maximum diameter of branches from the previous year’s growth. We also measured the number of branch tips (BTs in Roddy et al.^13^), in relation to branch diameter and length in 25 male and female apical branches of approximately 1 cm in diameter from site 1. For *A. cancellata* we measured the sex ratios of 325 individuals and measured the length of 5 needle leaves and nodal branch length from each of 25 individuals of each sex. For 62 individuals of each sex we measured basal diameter.

### Canopy characteristics

We also sampled 40 terminal branches of each sex for leaf and branch dimensions. To do this we removed all leaves between nodes from an apical branch per individual and noted leaf numbers and total leaf area per annual branch, as well as node length and stem diameter. We used these to determine mean leaf area and we dried leaves at room temperature to determine specific leaf area. Specific leaf area is correlated with many aspects of leaf physiology^30^. We measured leaf area with a LI-3000 Area Meter (LICOR, Lincoln, NE, USA) and leaves were dried for > 48 h at 70 °C in a drying oven to obtain dry mass. To get a measure of percentage seed set, we counted numbers of florets per single randomly collected inflorescence and numbers of seeds in a single cone on the same individual in each of 50 female individuals. We then dissected through a sample of 300 seeds from a cone from 32 individuals and used the visual presence of endosperm as indicative of a viable seed.

### Data analysis

All statistical analyses were conducted in R (R Core Team, 2021)^31^. To test whether the sexes were well mixed or not, we applied a runs test using the function RunsTest in the package ‘DescTools’ in R software on the sequence with which the sexes of *A. umbellata* were encountered along transects. To detect differences in male: female ratios we used a binomial test with 50:50 null expectation and to demonstrate competitive effects we used the Gini-coefficient on stem basal area by using the function gini.wtd in the package ‘dineq’ in R. This index of size inequality has long been used in plant competition^32,33^ and typically ranges from 0.2 (weak competition) to 0.7 (very strong competition). Differences in size between male and females were assessed using mixed effects models implemented with the function lmer in the package ‘lme4’ in R. These tests included site as a random factor. Linear models were constructed with the lm function in R and simplified using AIC scores in the package ‘olsrr’.

### Ethics declaration

Field studies on plants complied with relevant institutional, national, and international guidelines and legislation.
